# Mixed effects models but not t-tests or linear regression detect progression of apathy in Parkinson’s disease over seven years in a cohort: a comparative analysis

**DOI:** 10.1186/s12874-024-02301-7

**Published:** 2024-08-24

**Authors:** Anne-Marie Hanff, Rejko Krüger, Christopher McCrum, Christophe Ley, NCER-PD, Geeta Acharya, Gloria Aguayo, Myriam Alexandre, Muhammad Ali, Wim Ammerlann, Giuseppe Arena, Michele Bassis, Roxane Batutu, Katy Beaumont, Sibylle Béchet, Guy Berchem, Alexandre Bisdorff, Ibrahim Boussaad, David Bouvier, Lorieza Castillo, Gessica Contesotto, Nancy de Bremaeker, Brian Dewitt, Nico Diederich, Rene Dondelinger, Nancy E. Ramia, Angelo Ferrari, Katrin Frauenknecht, Joëlle Fritz, Carlos Gamio, Manon Gantenbein, Piotr Gawron, Laura georges, Soumyabrata Ghosh, Marijus Giraitis, Enrico Glaab, Martine Goergen, Elisa Gómez de Lope, Jérôme Graas, Mariella Graziano, Valentin Groues, Anne Grünewald, Gaël Hammot, Linda Hansen, Michael Heneka, Estelle Henry, Margaux Henry, Sylvia Herbrink, Sascha Herzinger, Alexander Hundt, Nadine Jacoby, Sonja Jónsdóttir, Jochen Klucken, Olga Kofanova, Pauline Lambert, Zied Landoulsi, Roseline Lentz, Laura Longhino, Ana Festas Lopes, Victoria Lorentz, Tainá M. Marques, Guilherme Marques, Patricia Martins Conde, Patrick May, Deborah Mcintyre, Chouaib Mediouni, Francoise Meisch, Alexia Mendibide, Myriam Menster, Maura Minelli, Michel Mittelbronn, Saïda Mtimet, Maeva Munsch, Romain Nati, Ulf Nehrbass, Sarah Nickels, Beatrice Nicolai, Jean-Paul Nicolay, Fozia Noor, Clarissa P. C. Gomes, Sinthuja Pachchek, Claire Pauly, Laure Pauly, Lukas Pavelka, Magali Perquin, Achilleas Pexaras, Armin Rauschenberger, Rajesh Rawal, Dheeraj Reddy Bobbili, Lucie Remark, Ilsé Richard, Olivia Roland, Kirsten Roomp, Eduardo Rosales, Stefano Sapienza, Venkata Satagopam, Sabine Schmitz, Reinhard Schneider, Jens Schwamborn, Raquel Severino, Amir Sharify, Ruxandra Soare, Ekaterina Soboleva, Kate Sokolowska, Maud Theresine, Hermann Thien, Elodie Thiry, Rebecca Ting Jiin Loo, Johanna Trouet, Olena Tsurkalenko, Michel Vaillant, Carlos Vega, Liliana Vilas Boas, Paul Wilmes, Evi Wollscheid-Lengeling, Gelani Zelimkhanov

**Affiliations:** 1 Transversal Translational Medicine, Luxembourg Institute of Health, Strassen, Luxembourg; 2 Translational Neurosciences, Luxembourg Centre for Systems Biomedicine, University of Luxembourg, Esch-Sur-Alzette, Luxembourg; 3 Department of Epidemiology, CAPHRI Care and Public Health Research Institute, Maastricht University Medical Centre+, Maastricht, The Netherlands; 4 Department of Nutrition and Movement Sciences, NUTRIM School of Nutrition and Translational Research in Metabolism, Maastricht University Medical Centre+, Maastricht, The Netherlands; 5 Parkinson Research Clinic, Centre Hospitalier du Luxembourg, Luxembourg, Luxembourg; 6 Department of Mathematics, University of Luxembourg, Esch-Sur-Alzette, Luxembourg

**Keywords:** Cohort studies, Epidemiology, Disease progression, Parkinson, Lost to follow-up, Statistical model

## Abstract

**Introduction:**

While there is an interest in defining longitudinal change in people with chronic illness like Parkinson’s disease (PD), statistical analysis of longitudinal data is not straightforward for clinical researchers. Here, we aim to demonstrate how the choice of statistical method may influence research outcomes, (e.g., progression in apathy), specifically the size of longitudinal effect estimates, in a cohort.

**Methods:**

In this retrospective longitudinal analysis of 802 people with typical Parkinson’s disease in the Luxembourg Parkinson's study, we compared the mean apathy scores at visit 1 and visit 8 by means of the paired two-sided t-test. Additionally, we analysed the relationship between the visit numbers and the apathy score using linear regression and longitudinal two-level mixed effects models.

**Results:**

Mixed effects models were the only method able to detect progression of apathy over time. While the effects estimated for the group comparison and the linear regression were smaller with high *p*-values (+ 1.016/ 7 years, *p* = 0.107, -0.056/ 7 years, *p* = 0.897, respectively), effect estimates for the mixed effects models were positive with a very small *p*-value, indicating a significant increase in apathy symptoms by + 2.345/ 7 years (*p* < 0.001).

**Conclusion:**

The inappropriate use of paired t-tests and linear regression to analyse longitudinal data can lead to underpowered analyses and an underestimation of longitudinal change. While mixed effects models are not without limitations and need to be altered to model the time sequence between the exposure and the outcome, they are worth considering for longitudinal data analyses. In case this is not possible, limitations of the analytical approach need to be discussed and taken into account in the interpretation.

**Supplementary Information:**

The online version contains supplementary material available at 10.1186/s12874-024-02301-7.

## Background

In longitudinal studies: “an outcome is repeatedly measured, i.e., the outcome variable is measured in the same subject on several occasions.” [1]. When assessing the same individuals over time, the different data points are likely to be more similar to each other than measurements taken from other individuals. Consequently, the application of special statistical techniques is required, which take into account the fact that the repeated observations of each subject are correlated [1]. Parkinson’s disease (PD) is a heterogeneous neurodegenerative disorder resulting in a wide variety of motor and non-motor symptoms including apathy, defined as a disorder of motivation, characterised by reduced goal-directed behaviour and cognitive activity and blunted affect [2]. Apathy increases over time in people with PD [3]. Specifically, apathy has been associated with the progressive denervation of ascending dopaminergic pathways in PD [4, 5] leading to dysfunctions of circuits implicated in reward-related learning [5].

## Methods

T-tests are often misused to analyse changes over time [6]. Consequently, we aim to demonstrate how the choice of statistical method may influence research outcomes, specifically the size and interpretation of longitudinal effect estimates in a cohort. Thus, the findings are intended for illustrative and educational purposes related to the statistical methodology. In a retrospective analysis of data from the Luxembourg Parkinson's study, a nation-wide, monocentric, observational, longitudinal-prospective dynamic cohort [7, 8], we assess change in apathy using three different statistical approaches (paired t-test, linear regression, mixed effects model). We defined the following target estimand: In people diagnosed with PD, what is the change in the apathy score from visit 1 to visit 8? To estimate this change, we formulated the statistical hypothesis as follows:

$$\text{H}0 :\text{ Mean change from visit }1\text{ to visit }8= 0$$

$$\text{HA }:\text{ Mean change from visit }1\text{ to visit }8 \ne 0$$

While apathy was the dependent variable, we included the visit number as an independent variable (linear regression, mixed effects model) and as a grouping variable (paired t-test). The outcome apathy was measured by the discrete score from the Starkstein apathy scale (0 – 42, higher = worse) [9], a scale recommended by the Movement Disorders Society [10]. This data was obtained from the National Centre of Excellence in Research on Parkinson's disease (NCER-PD). The establishment of data collection standards, completion of the questionnaires at home at the participants’ convenience, mobile recruitment team for follow-up visits or standardized telephone questionnaire with a reduced assessment were part of the efforts in the primary study to address potential sources of bias [7, 8]. Ethical approval was provided by the National Ethics Board (CNER Ref: 201,407/13). We used data from up to eight visits, which were performed annually between 2015 and 2023. Among the participants are people with typical PD and PD dementia (PDD), living mostly at home in Luxembourg and the Greater Region (geographically close areas of the surrounding countries Belgium, France, and Germany). People with atypical PD were excluded. The sample at the date of data export (2023.06.22) consisted of 802 individuals of which 269 (33.5%) were female. The average number of observations was 3.0. Fig. S1 reports the numbers of individuals at each visit while the characteristics of the participants are described in Table 1.

**Table 1:** Characteristics of the participants

Variables	Mean (SD) / n (%)	Min. – Max	Median (Pct25-75)	Missing N (%)
Age (years)	67.1 (10.9)	22.0 – 92.9	68.2 (60.2 – 74.6)	1 (0.1%)
Female sex	270 (33.7%)			0 (0.0%)
Years of education	13.0 (4.1)	1.0 – 30.0	13.0 (10.0 – 16.0)	9 (1.1%)
Actual diagnosis				0 (0.0%)
Parkinson’s disease	707 (88.2%)			
Parkinson’s disease dementia	95 (11.8%)			
Age at diagnosis (years)	62.4 (11.7)	18.0 – 91.0	63.0 (54.0 – 71.0)	8 (1.0%)
Years since diagnosis	5.0 (5.1)	0.0 – 32.3	3.2 (1.1 – 7.4)	54 (6.7%)
Apathy score (0 – 42)^﻿a^	12.0 (5.9)	1 – 36	13.0 (10.0 – 17.0)	54 (6.7%)
MDS-UPDRS I (0 – 52)^a^	10.4 (6.9)	0.0 – 39.0	9.0 (5.0—14.0)	33 (4.1%)
MDS-UPDRS II (0 – 52)^a^	11.0 (8.4)	0.0 – 48.0	9.0 (5.0—15.0)	24 (3.0%)
MDS-UPDRS III (0 – 132)^a^	34.1 (16.7)	0.0 – 100.0	32.0 (22.0—44.0)	21 (2.6%)
MDS-UPDRS IV (0 – 24)^a^	1.6 (3.2)	0.0 – 16.0	0.0 (0.0—1.0)	17 (2.2%)

^a^Greater = Worse

MDS-UPDRS: Movement Disorders Society – Unified Parkinson’s Disease Rating Scale [11], MDS-UPDRS I: non-motor symptoms, MDS-UPDRS II: patient-reported motor symptoms, MDS-UPDRS III: clinician assessed motor symptoms, MDS-UPDRS IV: motor complications

**Fig. 1:**
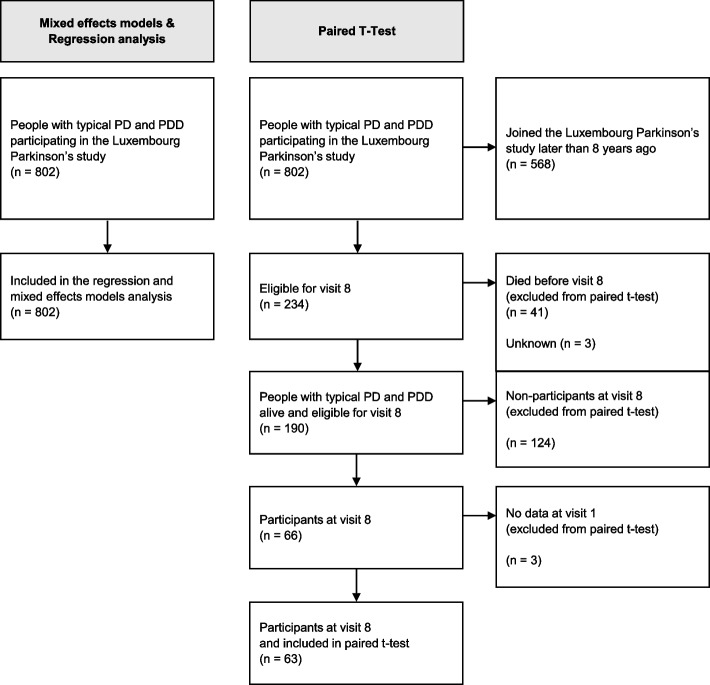
Flow diagram of patient recruitment

As illustrated in the flow diagram (Fig. 1), the sample analysed from the paired t-test is highly selective: from the 802 participants at visit 1, the t-test only included 63 participants with data from visit 8. This arises from the fact that, first, we analyse the dataset from a dynamic cohort, i.e., the data at visit 1 were not collected at the same time point. Thus, 568 of the 802 participants joined the study less than eight years before, leading to only 234 participants eligible for the eighth yearly visit. Second, after excluding non-participants at visit 8 due to death (*n* = 41) and other reasons (*n* = 130), only 63 participants at visit 8 were left. To discuss the selective study population of a paired t-test, we compared the characteristics (age, education, age at diagnosis, apathy at visit 1) of the remaining 63 participants at visit 8 (included in the paired t-test) and the 127 non-participants at visit 8 (excluded from the paired t-test) [12].

The paired two-sided t-test compared the mean apathy score at visit 1 with the mean apathy score at the visit 8. We attract the reader’s attention to the fact that this implies a rather small sample size as it includes only those people with data from the first and 8th visit. The linear regression analysed the relationship between the visit number and the apathy score (using the “stats” package [13]), while we performed longitudinal two-level mixed effects models analysis with a random intercept on subject level, a random slope for visit number and the visit number as fixed effect (using the “lmer”-function of the “lme4”-package [14]). The latter two approaches use all available data from all visits while the paired t-test does not. We illustrated the analyses in plots with the function “plot_model” of the R package sjPlot [15]. We conducted data analysis using R version 3.6.3 [13] and the R syntax for all analyses is provided on the OSF project page (https://doi.org/10.17605/OSF.IO/NF4YB).

## Results

**Fig. 2:**
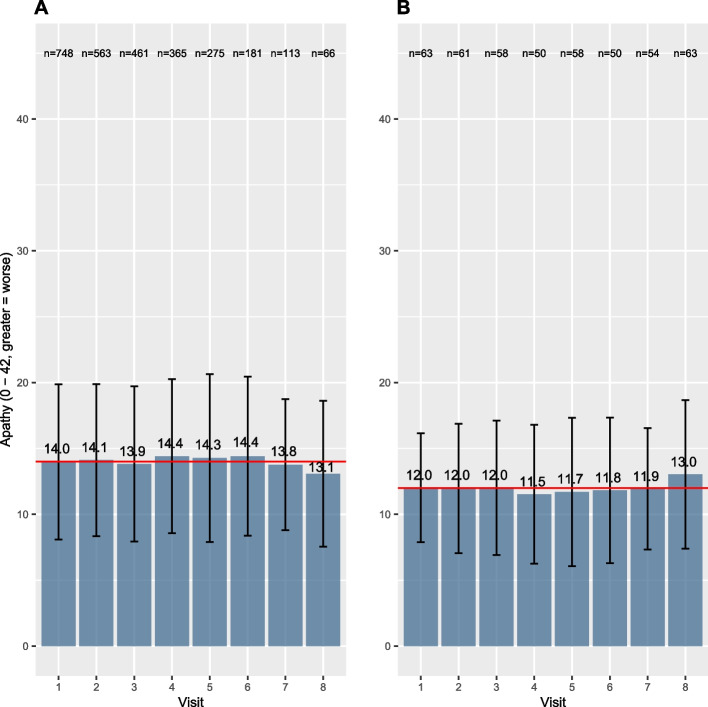
Bar charts illustrating apathy scores (means and standard deviations) per visit (Panel A: all participants, Panel B: subgroup analysed in the t-test). The red line indicates the mean apathy at visit 1

**Fig. 3:**
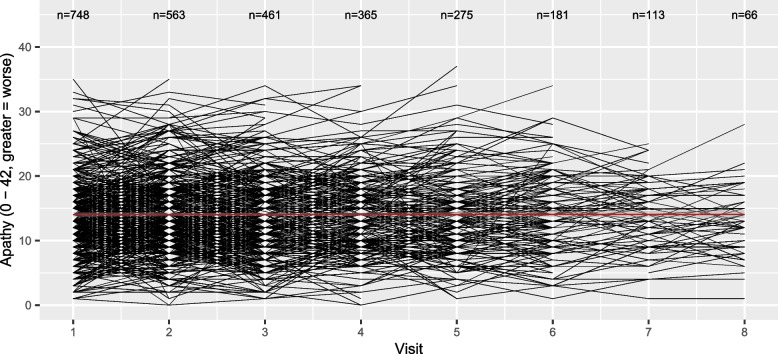
Scatterplot illustrating the individual trajectories. The red line indicates the regression line

Panel A in Fig. 2 illustrates the means and standard deviations of apathy for all participants at each visit, while the flow-chart (Fig. S1) illustrates the number of participants at each stage. On average, we see lower apathy scores at visit 8 compared to visit 1 (higher score = worse). By definition, the paired t-test analyses pairs, and in this case, only participants with complete apathy scores at visit 1 and visit 8 are included, reducing the total analysed sample to 63 pairs of observations. Consequently, the t-test compares mean apathy scores in a subgroup of participants with data at both visits leading to different observations from Panel A, as illustrated and described in Panel B: the apathy score has increased at visit 8, hence symptoms of apathy have worsened. The outcome of the t-test along with the code is given in Table 2. Interestingly, the effect estimates for the increase in apathy were not statistically significant (+ 1.016 points, 95%CI: -0.225, 2.257, *p* = 0.107). A possible reason for this non-significance is a loss of statistical power due to a small sample size included in the paired t-test. To visualise the loss of information between visit 1 and visit 8, we illustrated the complex individual trajectories of the participants in Fig. 3. Moreover, as described in Table S1 in the supplement, the participants at visit 8 (63/190) analysed in the t-test were inherently significantly different compared to the non-participants at visit 8 (127/190): they were younger, had better education, and most importantly their apathy scores at visit 1 were lower. Consequently, those with the better overall situation kept coming back while this was not the case for those with a worse outcome at visit 1, which explains the observed (non-significant) increase. This may result in a biased estimation of change in apathy when analysed by the compared statistical methods.

**Table 2:** Results from the group comparison, the linear regression and the linear mixed models

Statistical test	Change from visit 1 to visit 8	95% CI	*p*-value
Paired t-test	+ 1.016	-0.225, 2.257	0.107
Linear regression	-0.064	-0.856, 0.979	0.897
Linear mixed effects models	+ 2.680	1.880, 3.472	< 0.001

**Fig. 4:**
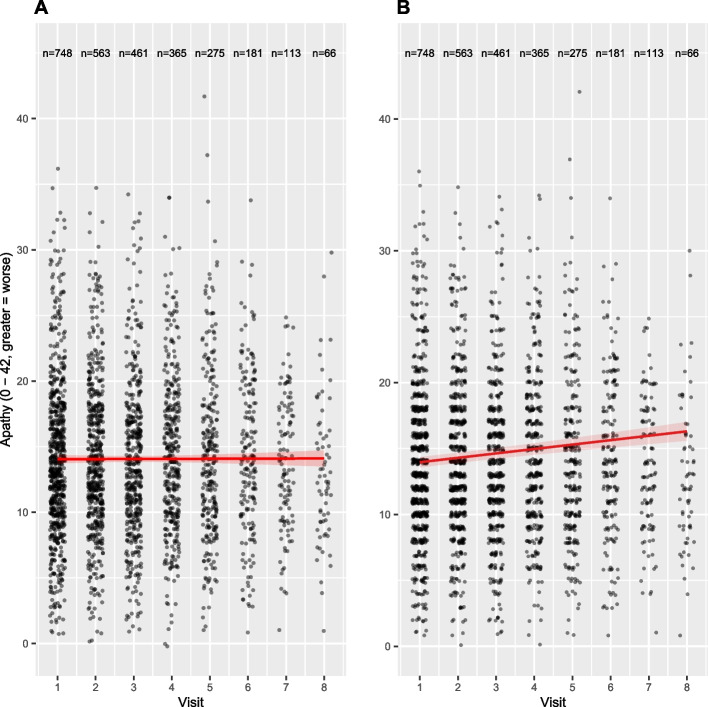
Scatterplot illustrating the relationship between visit number and apathy. Apathy measured by a whole number interval scale, jitter applied on x- and y-axis to illustrate the data points (Panel A: Linear regression, Panel B: Linear mixed effects model). The red line indicates the regression line

From the results in Table 2, we see that the linear regression coefficient, representing change in apathy symptoms per year, is not significantly different from zero, indicating no change over time. One possible explanation is the violation of the assumption of independent observations for linear regressions. On the contrary, the effect estimates for the linear mixed effects models indicated a significant increase in apathy symptoms from visit 1 to visit 8 by + 2.680 points (95%CI: 1.880, 3.472, *p* < 0.001). Consequently, mixed effects models were the only method able to detect an increase in apathy symptoms over time and choosing mixed effect models for the analysis of longitudinal data reduces the risk of false negative results. The differences in the effect sizes are also reflected in the regression lines in Panel A and B of Fig. 4.

## Discussion

The effect sizes differed depending on the choice of the statistical method. Thus, the paired t-test and the linear regression resulted in an output that would lead to different interpretations than the mixed effects models. More specifically, compared to the t-test and linear regression (which indicated non-significant changes in apathy of only + 1.016, -0.064 points from visit 1 to visit 8, respectively), the linear mixed effects models found an increase of + 2.680 points from visit 1 to visit 8 on the apathy scale. This increase is more than twice as high as indicated by the t-test and suggests linear mixed models is a more sensitive approach to detect meaningful changes perceived by people with PD over time.

Mixed effects models are a valuable tool in longitudinal data analysis as these models expand upon linear regression models by considering the correlation among repeated measurements within the same individuals through the estimation of a random intercept [1, 16, 17]. Specifically, to account for correlation between observations, linear mixed effects models use random effects to explicitly model the correlation structure, thus removing correlation from the error term. A random slope in addition to a random intercept allows both the rate of change and the mean value to vary by participant, capturing individual differences. This distinguishes them from group comparisons or standard linear regressions, in which such explicit modelling of correlation is not possible. Thus, the linear regression not considering correlation among the repeated observations leads to an underestimation of longitudinal change, explaining the smaller effect sizes and insignificant results of the regression. By including random effects, linear mixed effects models can better capture the variability within the data.

Another common challenge in longitudinal studies is missing data. Compared to the paired t-test and regression, the mixed effects models can also include participants with missing data at single visits and account for the individual trajectories of each participant as illustrated in Fig. 2 [18]. Although multiple imputation could increase the sample size, those results need to be interpreted with caution in case the data is not missing at random [18, 19]. Note that we do not further elaborate here on this topic since this is a separate issue to statistical method comparison. Finally, assumptions of the different statistical methods need to be respected. The paired t-test assumes a normal distribution, homogeneity of variance and pairs of the same individuals in both groups [20, 21]. While mixed effects models don’t rely on independent observations as it is the case for linear regression, all other assumptions for standard linear regression analysis (e.g., linearity, homoscedasticity, no multicollinearity) also hold for mixed effects model analyses. Thus, additional steps, e.g., check for linearity of the relationships or data transformations are required before the analysis of clinical research questions [17].

## Conclusion

While mixed effects models are not without limitations and need to be altered to model the time sequence between the exposure and the outcome [1], they are worth considering for longitudinal data analyses. Thus, assuming an increase of apathy over time [3], mixed effects models were the only method able to detect statistically significant changes in the defined estimand, i.e., the change in apathy from visit 1 to visit 8. Possible reasons are a loss of statistical power due to a small sample size included in the paired t-test and the violence of the assumption of independent observations for linear regressions. Specifically, the effects estimated for the group comparison and the linear regression were smaller with high *p*-values, indicating a statistically insignificant change in apathy over time. The effect estimates for the mixed effects models were positive with a very small *p*-value, indicating a statistically significant increase in apathy symptoms from visit 1 to visit 8 in line with clinical expectations. Mixed effects models can be used to estimate different types of longitudinal effects while an inappropriate use of paired t-tests and linear regression to analyse longitudinal data can lead to underpowered analyses and an underestimation of longitudinal change and thus clinical significance. Therefore, researchers should more often consider mixed effects models for longitudinal analyses. In case this is not possible, limitations of the analytical approach need to be discussed and taken into account in the interpretation.

### Supplementary Information

Supplementary Material 1.

## Data Availability

The LUXPARK database used in this study was obtained from the National Centre of Excellence in Research on Parkinson’s disease (NCER-PD). NCER-PD database are not publicly available as they are linked to the Luxembourg Parkinson’s study and its internal regulations. The NCER-PD Consortium is willing to share its available data. Its access policy was devised based on the study ethics documents, including the informed consent form approved by the national ethics committee. Requests for access to datasets should be directed to the Data and Sample Access Committee by email at request.ncer-pd@uni.lu. The code is available on OSF (10.17605/OSF.IO/NF4YB)

## Abbreviations

PD: Parkinson's disease
H0: Null hypothesis
HA: Alternative hypothesis
PDD: Parkinson's disease dementia
NCER-PD: National Centre of Excellence in Research on Parkinson's disease
OSF: Open Science Framework
i.e.: That is
n: Number
CI: Confidence Interval
