# Supplementary material for: Mixed effects models but not t-tests or linear regression detect progression of apathy in Parkinson’s disease over seven years in a cohort: a comparative analysis
Source: BMC Med Res Methodol. 2024 Aug 24;24:183. doi: 10.1186/s12874-024-02301-7 (PMC11344430; doi:10.1186/s12874-024-02301-7)
Supplement: Supplementary file 1 — Supplementary Material 1. [file 12874_2024_2301_MOESM1_ESM.docx]

Supplementary information for
Mixed effects models but not t-tests or linear regression detect progression of apathy in Parkinson’s disease over seven years in a cohort: A comparative analysis.

Anne-Marie Hanff1,2,3,4*(RN, MSN), Rejko Krüger(Dr)1,2,5 Christopher McCrum4(PhD), Christophe Ley6(PhD), on behalf of NCER-PD.

1 Transversal Translational Medicine, Luxembourg Institute of Health, Strassen, Luxembourg

2 Translational Neurosciences, Luxembourg Centre for Systems Biomedicine, University of Luxembourg, Esch-sur-Alzette, Luxembourg

3 Department of Epidemiology, CAPHRI Care and Public Health Research Institute, Maastricht University Medical Centre+, Maastricht, The Netherlands

4 Department of Nutrition and Movement Sciences, NUTRIM School of Nutrition and Translational Research in Metabolism, Maastricht University Medical Centre+, Maastricht, The Netherlands

5 Parkinson Research Clinic, Centre Hospitalier du Luxembourg, Luxembourg, Luxembourg

6 Department of Mathematics, University of Luxembourg, Esch-sur-Alzette, Luxembourg.

***Correspondence:**Anne-Marie Hanff
Mail: [anne-marie.hanff @ lih.lu](mailto:anne-marie.hanff@lih.lu)

*
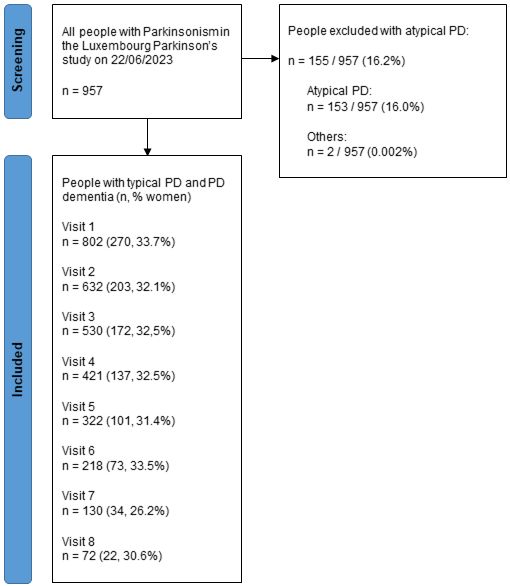

Figure S1: Flow diagram*

*Table S1: Comparison of characteristics between particpants at visit 8 (included in the paired t-test) and the non-participants at visit 8 (excluded from the paired t-test)*

| **Values at baseline** | **Participants without visit 8** (N = 127) | **Participants with visit 8** (N = 63) | **p-value** |
| --- | --- | --- | --- |
| **Apathy Score** | 14.3 (6.0) | 12.0 (4.1) | p = 0.003 |
| **Age (y.)** | 65.8 (11.7) | 62.3 (9.6) | p = 0.029 |
| **Age at diagnosis (y.)** | 59.5 (12.8) | 58.0 (10.3) | p = 0.367 |
| **Years of education** | 12.6 (3.6) | 14.0 (3.5) | p = 0.009 |

## 3. STROBE Reporting guideline – cohort studies ^7^

|  | | Item No | | Recommendation | Page No |
| --- | --- | --- | --- | --- | --- |
| **Title and abstract** | | 1 | | (*a*) Indicate the study’s design with a commonly used term in the title or the abstract | 1 |
|  |  |  |  | (*b*) Provide in the abstract an informative and balanced summary of what was done and what was found | 2 |
| **Introduction** | | | | | |
| Background/rationale | | 2 | | Explain the scientific background and rationale for the investigation being reported | 3 |
| Objectives | | 3 | | State specific objectives, including any prespecified hypotheses | 3-4 |
| **Methods** | | | | | |
| Study design | | 4 | | Present key elements of study design early in the paper | 3 |
| Setting | | 5 | | Describe the setting, locations, and relevant dates, including periods of recruitment, exposure, follow-up, and data collection | 4 |
| Participants | | 6 | | (*a*) Give the eligibility criteria, and the sources and methods of selection of participants. Describe methods of follow-up | 4-5 |
|  |  |  |  | (*b*) For matched studies, give matching criteria and number of exposed and unexposed | NA |
| Variables | | 7 | | Clearly define all outcomes, exposures, predictors, potential confounders, and effect modifiers. Give diagnostic criteria, if applicable | 4 |
| Data sources/ measurement | | 8* | | For each variable of interest, give sources of data and details of methods of assessment (measurement). Describe comparability of assessment methods if there is more than one group | 4 |
| Bias | | 9 | | Describe any efforts to address potential sources of bias | 7 |
| Study size | | 10 | | Explain how the study size was arrived at | 5-7 |
| Quantitative variables | | 11 | | Explain how quantitative variables were handled in the analyses. If applicable, describe which groupings were chosen and why | 4 |
| Statistical methods | | 12 | | (*a*) Describe all statistical methods, including those used to control for confounding | 7 |
|  |  |  |  | (*b*) Describe any methods used to examine subgroups and interactions | NA |
|  |  |  |  | (*c*) Explain how missing data were addressed | NA |
|  |  |  |  | (*d*) If applicable, explain how loss to follow-up was addressed |  |
|  |  |  |  | (*e*) Describe any sensitivity analyses | NA |
| **Results** | | | | |  |
| Participants | | 13* | | (a) Report numbers of individuals at each stage of study—eg numbers potentially eligible, examined for eligibility, confirmed eligible, included in the study, completing follow-up, and analysed | 6 |
|  |  |  |  | (b) Give reasons for non-participation at each stage | 6 |
|  |  |  |  | (c) Consider use of a flow diagram | 6 |
| Descriptive data | | 14* | | (a) Give characteristics of study participants (eg demographic, clinical, social) and information on exposures and potential confounders | 4-5 |
|  |  |  |  | (b) Indicate number of participants with missing data for each variable of interest | 4-5 |
|  |  |  |  | (c) Summarise follow-up time (eg, average and total amount) | 4-5 |
| Outcome data | | 15* | | Report numbers of outcome events or summary measures over time | 9 |
| Main results | 16 | | (*a*) Give unadjusted estimates and, if applicable, confounder-adjusted estimates and their precision (eg, 95% confidence interval). Make clear which confounders were adjusted for and why they were included | | 7-12 |
|  |  |  | (*b*) Report category boundaries when continuous variables were categorized | | NA |
|  |  |  | (*c*) If relevant, consider translating estimates of relative risk into absolute risk for a meaningful time period | | NA |
| Other analyses | 17 | | Report other analyses done—eg analyses of subgroups and interactions, and sensitivity analyses | | NA |
| **Discussion** | | | | | |
| Key results | 18 | | Summarise key results with reference to study objectives | | 12-13 |
| Limitations | 19 | | Discuss limitations of the study, taking into account sources of potential bias or imprecision. Discuss both direction and magnitude of any potential bias | | 13 |
| Interpretation | 20 | | Give a cautious overall interpretation of results considering objectives, limitations, multiplicity of analyses, results from similar studies, and other relevant evidence | | 12-13 |
| Generalisability | 21 | | Discuss the generalisability (external validity) of the study results | | NA |
| **Other information** | | | | | |
| Funding | 22 | | Give the source of funding and the role of the funders for the present study and, if applicable, for the original study on which the present article is based | | 15 |
